# Assessment of pre and postoperative symptomatology in patients undergoing inferior turbinectomy

**DOI:** 10.1016/S1808-8694(15)31201-5

**Published:** 2015-10-20

**Authors:** Adriano de Amorim Barbosa, Nelson Caldas, Alberto Xavier de Morais, Alexandre José da Costa Campos, Sílvio Caldas, Fábio Lessa

**Affiliations:** 1 Medical Residence program, specialist, director.; 2 Otorhinolaryngologist, Faculty Professor, Discipline of Otorhinolaryngology, HC-UFPE.; 3 Resident physician.; 4 Residence physician.; 5 Otorhinolaryngologist, Joint Professor and Head of the Discipline of ORL HC-UFPE.; 6 Speech therapist and audiologist. Discipline of Otorhinolaryngology, Hospital das Clínicas, Federal University of Pernambuco.

**Keywords:** rhinitis, turbinectomy, obstruction nasal

## Abstract

Partial inferior turbinectomy is a procedure directed to treat nasal obstruction secondary to hypertrophy rhinitis. This study evaluates the impact of this procedure in the quality of life of the patients, analyzing the improvement of other symptoms such as rhinorrhea, nasal itching and sneezing after six months of the surgical procedure. **Study design:** clinical prospective. **Material and Method**: Forty-nine patients submitted to turbinectomy associated or not with septoplasty, received questionnaires to grade the intensity of symptoms. Through the comparison of severity of symptoms before and after six months of the surgery, it was possible to evaluate the degree of improvement of each symptom. The results were classified as null, good, moderate and great, and we subtracted postoperative score from the preoperative score. **Results**: The nasal obstruction presented good or great results in 98% of the patients. For rhinorrhea, the surgery has resulted in good or great improvement in 49% of the cases. Sneezing presented good or great results in 81.6% of the patients and, nasal itching, 45%. **Conclusion**: The study showed that the clinical benefits obtained with the partial inferior turbinectomy are not limited to nasal obstruction, extending also to others symptoms of rhinitis, mainly in relation to sneezing crisis.

## INTRODUCTION

Allergic rhinitis is syndrome characterized by symptoms such as nasal congestion, rhinorrhea, nasal itching, and sneezing^1^. These symptoms result from chronic nasal mucosa inflammation of allergic origin and may be followed by other symptoms such as eye, roof of the mouth and throat itching^2^. Allergic rhinitis does not have gender preference and affects people of all ages, although it is more frequently observed among young patients.

Diagnosis is essentially clinical, and may be complemented by skin prick tests or serum assays for allergen-specific IgE^2^ .

Treatment of allergic rhinopathy should be based on environmental control, while patient should avoid contact with potential allergens and medicines. Several drugs, such as antihistamines, corticosteroids, chromoglycate, anticholinergic and leukotriene modifiers may act to control crises^2^. Choice of medicine should be based on symptoms’ quality and intensity observed in the patient. Surgical treatment is indicated to patients with important nasal obstruction non-responsive to clinical therapy^3^.

Partial inferior turbinectomy is a surgical procedure traditionally adopted for nasal de-obstruction of patients with allergic rhinopathy non-responsive to clinical treatment. This procedure consists of partial resection of the lower nasal turbinate bones^3^, ^4^ . Former literature studies reporting turbinectomy date from 1908 with Escat.^1^ In 1920 and on, after Citelli presented this surgical technique to treat nasal obstruction, it was disseminated and many other related studies were published in the following decades. Labayle, in 1949, called the attention to the physiological role of the nasal conchae and described turbinectomy of the bone submucosa. In 1951, House praised distinct surgical procedures for nasal conchae of major bones and fine mucosa, as well as for conchae of minor bones and great mucosa. Missaka, in 1972, by means of nasal biopsies in patients submitted to turbinectomy, described partial recovery of nasal mucous structures after 6 months of surgery.^1^ Today, this is the most frequent procedure in otorhinolaryngology’s routine, and is recognized as an effective treatment for nasal obstruction secondary to hypertrophic rhinitis.^5^

The main complications of turbinectomy are trans and post-operative hemorrhage, formation of nasal synechiae, abnormal nasal sensation, nasal scabs and atrophic rhinitis^1^, ^6^, ^7^.

Although it is considered an effective treatment for nasal obstruction, we have found only few reports in the literature evaluating the impact of this procedure over other symptoms present in patients with allergic rhinitis such as rhinorrhea, sneezing and nose itching.

The purpose of this study was to assess improvement of allergic rhinitis patients presenting nasal obstruction non-responsive to clinical treatment, sneezing and nasal itching six months after being submitted to partial inferior turbinectomy.

## MATERIAL AND METHODS

The study comprised 49 patients assisted at the Otorhinolaryngology Ambulatory of *Hospital das Clínicas, Federal University of Pernambuco* (HC-UFPE) with clinical diagnosis of allergic rhinitis presenting nasal obstruction non-responsive to clinical treatment, environmental control and use of topic corticosteroids. All patients were submitted to surgical treatment (partial bilateral inferior turbinectomy with or without septoplasty) in the period of 2000 and 2002. Patients’ ages ranged from 12 to 61 years (mean age of 25), among which 25 were men and 24 were women. In the preoperative period, after signing the compliance term, patients answered a survey to rate severity level of nasal obstruction, rhinorrhea, nose itching, and sneezing as null (grade 0), mild (grade 1), moderate (grade 2) and severe (grade 3).

Surgery was performed by the same surgeon and under general anesthesia. It was classical turbinectomy including simple resection of mucous or bone excess of the lower nasal conchae followed by careful monopolar cauterization of occasional bleeding spots. When necessary, septoplasty was performed through Cottle’s technique. Out of 49 patients, 22 were submitted to turbinectomy and septoplasty, while 27 underwent turbinectomy alone. In all cases, nasal packing and gauze were placed and removed 24 hours later. Postoperatively, patients were guided to do nasal washes with isotonic saline solutions and not to use drugs that could interfere with outcomes, such as corticoids and antihistamines. Revisions were made every 3-4 days for secretion and aspirations of clots, scabs and fibrin removal until complete tissue healing was reached.

Six months after surgery and following the previous criteria, patients answered another survey to update information on severity of symptoms. Comparison between intensity of symptoms observed pre and postoperatively allowed us to determine improvement or worsening of the clinical state. We subtracted postoperative score from the preoperative score concerning symptoms severity, and categorized the results as 0 = null; 1 = fair; 2 = good; and 3 = excellent.

## RESULTS

**Graph 1:**
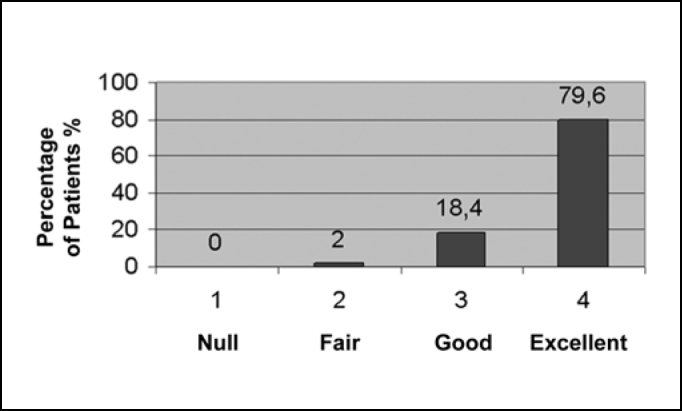
Response to nasal obstruction after 6 months of surgery.

**Graph 2:**
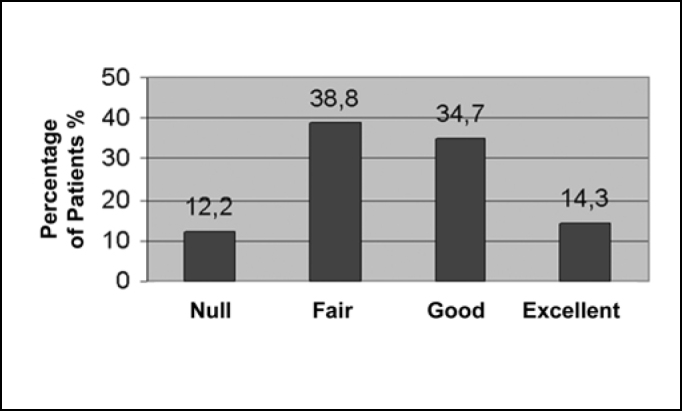
Response to rhinorrhea after 6 months of surgery.

**Graph 3:**
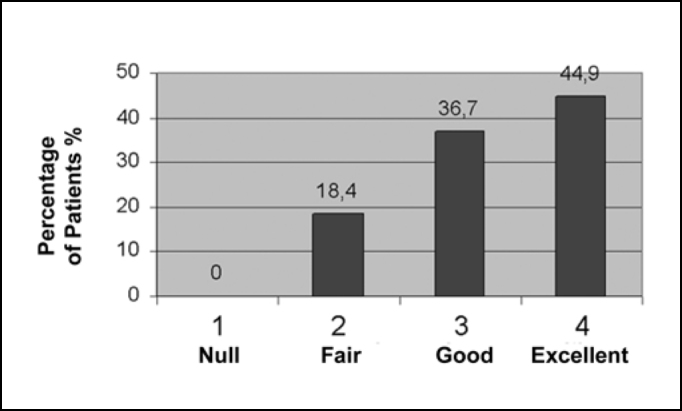
Response to sneezing after 6 months of surgery.

**Graph 4:**
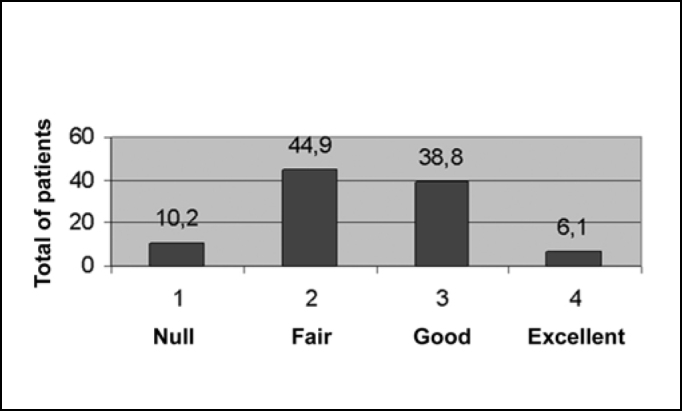
Response do nasal itching after 6 months of surgery.

All symptoms presented clinical improvement, while every outcome was statistically significant with p<0.05. Relative to nasal obstruction, the results in 39 patients (79.6%) were excellent, in 9 (18.4%) were good, while only in 1 (2%) was fair and in none it was null (Graph 1). Regarding rhinorrhea, 7 patients (14.3%) showed excellent improvement, 17 (34.7%) good, 19 (38.8%) fair and 6 (12.2%) null, as can be observed in Graph 2. Results concerning sneezing were excellent in 22 (44.9%) patients, good in 18 (36.7%) and fair in 9 (18.4%), and, similarly to nasal congestion, no patient had null improvement (Graph 3). Finally, concerning nose itching, only 3 patients (6.1%) had excellent improvement of this symptom, 19 (38.8%) had good, 22 (44.9%) had fair and 5 (10.2%) had not noticed any changes (Graph 4).

## DISCUSSION

Clinical manifestation of allergic rhinitis is through runny and itchy nose, sneezing and nasal congestion for which treatment is essentially clinical. Patients with obstructive symptoms who do not improve with clinical treatment may be benefited by surgery^2^, ^5^, ^8^, ^11^. Among surgical options, partial inferior turbinectomy is considered an effective therapeutic choice for nasal obstruction secondary to hypertrophic rhinitis, although this procedure is poorly studied and the benefits on other symptoms of allergic rhinitis have not been established so far.

The use of surveys to better understand the importance of chronic symptoms in patients’ lives is well-recognized by all medical fields, and they are a very effective research tool for assessment of clinical and surgical outcomes and their impact over the patients’ lives.

Ophir et al.^12^ studied 186 patients using surveys and performing previous rhinoscopy 10 to 15 years after they were submitted to turbinectomy. They demonstrated that there was improvement in nasal obstruction in 82% of patients, while there were no abnormalities in previous rhinoscopy in 88% of them. Rhinorrhea persisted in 34% of patients, out of which 19% used topic or systemic medications to control runny nose.

Mori et al.^13^ followed 45 patients with allergic rhinitis who were submitted to submucous turbinectomy during 3 to 5 years after surgery. They observed that, after this procedure, there was significant improvement of nasal obstruction and sneezing: 68.9% responded well after 3 years and 73.3% after 5 years. Regarding sneezing, 44.4% of patients had good response after 3 years, while 33.3% after 5 years. Improvement of rhinorrhea was lower: 43.9% of patients after 1 year of surgery, 17.8% after 3 years, and 3.4% after 5 years.

In the present study, the symptoms that responded better to surgery were also nasal obstruction and sneezing, with 98% and 81.6% of good and excellent results, respectively. Similarly to Mori et al.^13^ and Ophir et al.^12^ observations, results were more limited in rhinorrhea, where more than half of the cases (51%) presented null or fair improvement. The worst results in our study were in nose itching, which presented good or excellent responses in 45% of patients only. However, even for those symptoms, it is evident that there was improvement for the great majority of cases (87.8% for rhinorrhea and 89.8% for itching). No major complications, atrophic changes or purulent infections of the nasal mucosa were observed in the patients. Concerning nasal obstruction, it is important to emphasize that some of these patients had also been submitted to septoplasty and, therefore, good outcomes should also be attributed to this procedure, which, in otolaryngology routine and in the majority of this type of patient, is usually performed in association with turbinectomy. However, outcomes assessment in nasal obstruction was not the main objective of this study – once this benefit with turbinectomy is already largely established in literature -, but rather the response of other symptoms to this surgical procedure. The major purpose of partial inferior turbinectomy is to allow better airway flow through nasal fossae. However, once turbinectomy involves partial resection of the lower turbinate bones which imperatively reduces nasal mucosa surface exposed to action of allergens and other stimuli, as well as the volume of glandular tissue of this region, this could also justify the improvement of other allergic rhinitis symptoms observed in the present study.

In this research study, improvement was observed not only in nasal obstruction, but also in other symptoms studied, at a higher or lower level, after six months of surgery, although limited to rhinorrhea and nose itching. These results demonstrated that the benefits obtained with partial inferior turbinectomy are not restricted to improvement of nasal obstruction, but rather better outcomes can be expected concerning other symptoms, specially sneezing.

## CLOSING REMARKS

The present study shows that partial bilateral inferior turbinectomy is an effective procedure to lead not only to improvement of nasal obstruction, but also to benefit other symptoms of allergic rhinitis, particularly in sneezing and nose running and itching.
